# More than Meets the Eye: Understanding Perceptions of China Beyond the Favorable–Unfavorable Dichotomy

**DOI:** 10.1007/s12116-021-09320-1

**Published:** 2021-02-24

**Authors:** Xiaojun Li

**Affiliations:** The University of British Columbia, Vancouver, Canada

**Keywords:** Public opinion, Misinformation, Foreign direct investment, China, Canada

## Abstract

How is China viewed by citizens of other countries? Popular polling data based on the feeling thermometer scale can reveal overall patterns of public sentiment toward China, but they do not necessarily capture the multidimensional preferences of the public. This article takes a deeper dive into a series of surveys conducted in Canada that covered a wide range of topics, from trade and investment to international leadership. Two broad conclusions follow. First, public perceptions of China are much more nuanced and conflicted than can be quickly gleaned from the simple dichotomy of “favorable versus unfavorable,” especially as one moves from overall impressions to more specific policy issues. Second, misperceptions of China are widespread and may be difficult to overcome, especially among those who already view China negatively. At a time when countries around the world are grappling with the rise of China and its expanding global footprint, failure to account for these features in public opinion about China may lead to misguided policies.

**Supplementary Information:**

The online version contains supplementary material available at 10.1007/s12116-021-09320-1.

## Introduction

On April 21, 2020, Pew Research Center released their latest survey on Americans’ views of China. One of the most striking findings from the survey was that nearly two-thirds (66%) of Americans now hold an unfavorable opinion of China, over twice as many as those who said their opinion was favorable (26%). The numbers mark a steep jump of nearly 20% from just two years ago and are the highest ever recorded by Pew since this question was first asked back in 2005, when only 35% of Americans said their opinion of China was negative.

With headlines like “Americans take an ever more negative view of China” (The Economist 2020), “Americans’ opinion of China nosedives amid coronavirus pandemic” (Steinhauser 2020), and “China’s unfavorability in the USA jumps to the highest level recorded” (Dibble 2020), news outlets quickly picked up the story, which was widely circulated in social media almost instantaneously. Before long, politicians were jumping on the bandwagon as well. “China has long been our greatest geopolitical threat,” tweeted Senator Ted Cruz. “As we learn more about the Chinese Communist Party’s decision to suppress information that could have prevented the #CoronavirusPandemic, the American people are now seeing just how much destruction China is capable of.”

Does the rise in perceived negativity over China imply a shift in public preferences for tougher policies on Beijing? Politicians seem to think this is the case. President Donald Trump and his Democratic rival, President-Elect Joe Biden, began ramping up the anti-China rhetoric in their campaign ads, competing to “out-hawk” each other on Beijing (Khalid 2020). To boost their prospects of re-election, Republicans and Democrats alike were feeling the need to go on the record as criticizing China, with more than 250 China-related bills currently before Congress, advocating more restrictions and disengagement (Gillman 2020).

The realities are often more complex than can be distilled into a single binary affective measure of favorability. Public sentiments toward foreign countries are known to be finicky and sensitive to external shocks, such as the current COVID-19 pandemic, and can be further exacerbated by political rhetoric and the spread of misinformation and disinformation. In addition, individuals may have different emotional responses to various aspects of a foreign country, depending on their own experiences, ideology, and temperament, and thus, their policy preferences can be more varied as well.

The “China in the world” model outlined in the introduction to this special issue calls for better understanding of how the rest of the world views China and its rise (Fravel et al. 2021). The present article tackles this question by moving beyond popular public opinion polls that focus on a single measure of favorability, drawing instead on a series of public opinion surveys conducted in Canada that covered a wide range of issues over the course of 2 years during which Canada–China relations have reached both new highs and new lows. The results reveal that Canadian opinions of China have fluctuated in response to bilateral relations in predictable ways, dipping to the low twenties in terms of favorability after the diplomatic crisis following the arrest of Huawei CFO Meng Wanzhou. In the meantime, however, the public’s desire for continued economic exchange with China has remained strong. Asked to choose the top priority for the Canadian government, Canadians were consistent in their preferences, with expansion of trade and investments topping the list.

Despite a general willingness to see more economic exchange with China, Canadians were also wary of Chinese investments, ranking them lower than comparable ones from Japan, the Netherlands, and the USA. Part of this may have been driven by widely held misperceptions regarding Chinese investments in Canada. Canadians on average grossly overestimated the proportion of foreign direct investment (FDI) in Canada coming from China—believing it was over 10 times larger than the official statistic of 3%. Furthermore, nine out of ten were misinformed about how foreign investment rules and practices govern these investments, especially those from foreign state-owned firms. Correcting these misperceptions using official statistics and documents improved support for FDI projects from China to some extent, but the effects were moderated by overall perceptions of China.

Two broad conclusions follow. First, public perceptions of China are much more nuanced and conflicted than can be quickly gleaned from the simple dichotomy of “favorable versus unfavorable,” particularly as one moves from overall impressions to more specific policy issues. Second, misperceptions of China are widespread and may be difficult to overcome, especially among those who already view China negatively. At a time when countries around the world are grappling with the rise of China and its expanding global footprint, failure to account for these features in public opinion about China may lead to misguided policies.

## Gauging Public Perceptions of China

Scholars of international relations have largely come to the consensus that foreign policies are not only shaped by a country’s position in the international system, but also driven by forces operating within a society. A large body of literature has addressed questions on how the public thinks about foreign affairs, and how these public preferences may shape foreign policymaking (Kertzer 2018). In recent works, scholars have demonstrated the constraining effects of public opinion on the scope of foreign policy choices available to decision-makers (e.g., Baum and Potter 2015). Others have shown that popular support can empower national leaders to overcome institutional barriers to achieve foreign policy goals (e.g., Flores-Macías and Kreps 2013; Foyle 1999). This is certainly the case for democracies through mechanisms of democratic constraint and accountability (Casey 2008), but it can even apply in authoritarian countries such as China through mechanisms of public protest and quasi-institutions of accountability (Hao and Su 2005; Li and Chen 2020; Reilly 2012; Weiss 2013).

Given that public opinion in democracies is “at the heart of presidential decision making” (Hinckley 1992: 4), it is essential for policy makers to have a better understanding of public interests and preferences when drafting their policies on foreign countries. This is even more crucial for China, a country of such importance and yet perceived to be much different from the West. Tracing American images of and policies on China from 1776 to the present day, for example, Turner (2014) shows that American views and opinions of China have always been the central to the formulation, enactment, and justification of USA–China policy in Washington.

One way of gauging public sentiments on foreign countries is to measure it with a feeling thermometer. In public opinion polls, the feeling thermometer asks respondents to rank their views on a given subject, a country in this case, on a scale from “cold” (unfavorable) to “hot” (favorable), similar to the temperature scale of a real thermometer. The earliest thermometer-type question on China appeared in a Gallup poll in 1967, in which American respondents were asked about their “overall opinion” of China, along with a dozen other countries, using a four-point Likert scale from “very favorable” to “very unfavorable.” In that poll, over nine out of ten Americans held an unfavorable opinion of China. Since then, Gallup asked the same question on China twice in the 1970s, six times in the 1980s, ten times in the 1990s, and annually every February, starting in 2001. Another early example in the USA is the Chicago Council on Global Affairs, which has been conducting a nationwide public opinion survey on foreign policy since 1974. The survey includes a question asking respondents to evaluate foreign countries through a feeling thermometer ranging from 0 for a cold country to 100 for a hot one. China first appeared with 24 other countries in the 1982 version of the survey, when it scored 47 points on the thermometer (Schneider 1985).

Outside of the USA, a number of think tanks and non-profit organizations in Europe, Asia, and Africa have conducted similar surveys to track public perceptions of China. Funded by the German Marshall Fund, Transatlantic Trends is an annual survey of public opinion in 11 European Union countries plus Turkey and the USA. Between 2002 and 2014, the survey included a question to measure the respondent’s overall view of China using the same four-point scale as the Gallup polls. Similarly, the Genron NPO in Japan since 2005 has annually conducted opinion polls in Japan and China prior to the Tokyo Beijing Forum, which is the most influential Track II platform between the two countries. These surveys reveal the impressions held by each country’s citizens toward the other, among other things.

In Australia, the Lowy Institute since 2005 has conducted an annual “feelings thermometer” poll that measures Australians’ feelings towards a range of countries and territories. China has been a fixture in these polls, earning a generally favorable rating (> 50°) in all but the most recent one (49°) on a scale from 0° (coldest feelings) to 100° (warmest feelings) (Kassam 2019). The Afrobarometer also began probing perceptions of China in its 2014/2015 surveys, finding that the public in 36 African countries held generally favorable views of China and its economic and assistance activities (Lekorwe et al. 2016).

Perhaps, the most widely known and cited opinion polls of public perceptions of China are those conducted by Pew Research Center, which has been tracking global attitudes toward China since 2005. Unlike the other polls, which focus on public attitudes in one country or one region, the Pew Global Attitudes Survey is much more comprehensive, covering a wide range of countries around the world. In the most recent Global Attitudes Survey (December 2019), for example, a median of 40% across the 34 countries surveyed reported a favorable opinion of China, compared with a median of 41% with an unfavorable opinion (Silver et al. 2019). Beijing’s most positive perceptions came from Russia (71% favorable), Nigeria (70%), and Lebanon (68%), whereas the most negative views were found in Japan (85% unfavorable), Sweden (70%), and Canada (67%).

What can we learn from this increased amount of data on public perceptions of China? Scholars of public opinion research have long debated the ability of citizens to form rational and coherent opinions. On the one hand, skeptics question the knowledge of the general public, especially on foreign policy issues. Zaller (1992), for example, contends that individuals “do not typically carry around in their heads fixed attitudes, and instead respond on the basis of whatever considerations are most immediately salient in their minds” (Zaller 1992: 64). Supporters, on the other hand, argue that opinions examined over a sufficient span of time can reveal central tendencies of popular preferences. Page and Shapiro (1992), for example, conclude that the “collective policy preferences of the American public are predominantly *rational*, in the sense that they are *real* … *coherent* … and that when collective policy preferences change, they … do so in … *predictable* ways” (Page and Shapiro 1992: xi). Reconciling these two opposing views, Kuklinski and Quirk (2000) argue that while the assumption of a politically competent and rational public may be overly optimistic, ordinary citizens should do better when they opine on broader and longer-standing features of politics such as political parties, social groups, ideologies, and established leaders.

Public perceptions of China tracked over a long time seem to suggest that citizens in general are rational when it comes to evaluating foreign countries. This can be seen in historical data on American perceptions of China collected by the Gallup polls; views have swung back and forth over time, but the movement was largely in congruence with major events, both within China and in the bilateral relations between the two countries (Newport 1999). For example, after posting the lowest approval rating, in 1967, during the height of the Cold War, public perceptions of China gradually warmed up as the two countries normalized their diplomatic relations after Nixon’s visit in 1972. Public views had undergone a complete reversal by the honeymoon period of the mid-1980s, with over 65% of the US public having a favorable opinion of China in 1987. The pendulum moved in the opposite direction after the Tiananmen protests in June 1989, when favorable opinions of China nosedived from 72% in February to 34% in August while unfavorable opinions quadrupled from 13 to 54%. Over the next 10 years, American attitudes on China slowly recovered but tended to remain more unfavorable than favorable. In the last Gallup poll before the new millennium, conducted between May 7 and 9 in 1999 amidst Chinese outrage at the US bombing of the Chinese embassy in Belgrade, views on China broke strongly negative—56% unfavorable and only 38% favorable.

Looking at these approval ratings, one might ask: do public perceptions follow the United States’ China policies, or is it the other way around? There is no easy answer to this question. On the one hand, the US policy on China has always actively produced and reaffirmed China’s image in the eyes of the American public. On the other hand, the constraining effect of public opinion on foreign policy suggests that perceptions of China should contribute to the subsequent direction of America’s China policies. Regardless of the causal arrow, however, it is reasonable to conclude that public opinions should determine the boundaries of politically possible policy options.

**Fig. 1:**
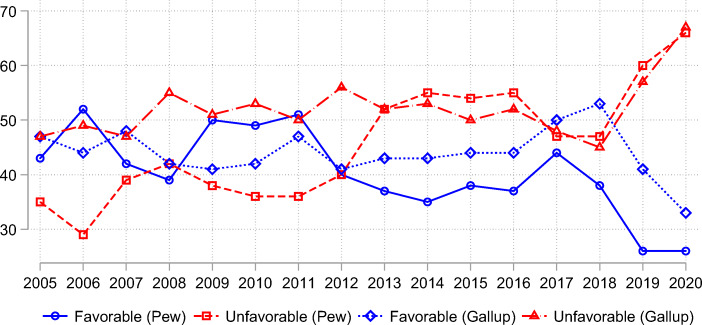
Americans’ favorability toward China, 2005–2020. Source: Gallup and Pew Research Center

While gauging public perceptions using the feeling thermometer can be informative for both policy makers and scholars, these measures have two major limitations. First, public sentiments about foreign countries can easily be swayed by external shocks. The dramatic shift in public perceptions of China in 1989 is a good example. Likewise, the recent upsurge in unfavorable views almost certainly can be largely attributed to the COVID-19 pandemic. The finicky nature of public perceptions is further problematized when we compare results from different polls. Figure 1 plots Americans’ favorability toward China from 2005 to 2020, using the two longest series of data from Gallup and Pew Research Center, respectively. A couple of patterns are worth noting. One is the large amount of discrepancy between the two polls conducted within the same year. The average absolute differences during this time are seven points for the approval ratings (14 points before 2001 and two points afterwards) and eight points for the disapproval ratings. The second pattern is that in terms of year-to-year changes over time during this period, the trends in the two polls do not necessarily move in tandem. In particular, the favorability ratings from the two polls moved in opposite directions more than half of the time (nine out of 15). The unfavorability ratings performed slightly better, moving in opposite directions in six out of 15 years. Hence, depending on the specific polls under examination, one can arrive at very different and even contradictory conclusions when evaluating American public perceptions of China.

Second, while the feeling thermometer is an easily interpreted measure of affect, with little ambiguity—warm indicates positive feelings and cold negative—the reality is often more complex. Depending on their particular experiences, ideology, and temperament, individuals may have vastly different emotional responses to various aspects of a foreign country. Evans and Li (2019), for example, find that interactions with Chinese people, knowledge of Chinese culture and history, and awareness of China’s economic dynamism all have a positive influence on Canadian views of China, whereas China’s investments in Canada, its environmental policies, and especially its system of government and human rights record are all major sources of disapproval. Similarly, in the 2016 Lowy Institute poll, Australians were found to have favorable views about China’s people, culture, history, and economic growth, but negative views on China’s environmental policies, system of government, military activities, and human rights record (Oliver 2016). By extension, the public’s policy preferences on China can be varied—for example, a person can be highly critical of China’s human rights record but at the same time be open to closer economic ties, which would imply a preference for a combination of policies that vary on issues rather than on outright engagement or containment.

The above discussion suggests that public views on China could be much more nuanced than can be quickly gleaned from the simple dichotomy of “favorable versus unfavorable.” To further illustrate this point, in the remainder of the article, I report findings from a series of public opinion surveys conducted in Canada over the course of 2 years, between 2017 and 2019. The next section will briefly review Canada–China relations from 1949 to the present day to provide context for the three surveys. The subsequent two sections will use the survey data to examine public perceptions of China in Canada beyond the feeling thermometer measures.

## Canadian Views of China: Context and Survey Design

In the first 20 years after the founding of the People’s Republic, Canada’s engagement with China was limited. Policy makers in Ottawa treated the Far East country as either an intractable Cold War problem or a market for Canadian wheat. The formalization of relations under Pierre Elliott Trudeau’s government on October 13, 1970, helped bring China into greater contact with the international community and paved the way for the thawing of the USA–China relations 2 years later. Increased exchanges between the two countries on trade and investment during China’s reform era in the 1990s solidified a special bilateral relationship, with former Chinese Premier Zhu Rongji praising Canada as China’s “best friend;” these developments culminated in an agreement to build a “strategic partnership,” announced during former President Hu Jintao’s official visit to Canada in 2005.

Canada’s engagement with China was inconsistent during the Harper years, from 2006 to 2015. Prioritizing human rights and security issues in dealings with China, Harper personally hosted the Dalai Lama in 2007 and snubbed the Beijing Olympics in 2008, while his administration amended the Investment Canada Act to restrict takeovers of Canadian firms by foreign state-owned enterprises (SOEs) after the China National Offshore Oil Corporation controversially purchased Canadian oil and gas company Nexen. However, under rising pressure from the business community to deepen ties with China, the bilateral relationship returned to a positive footing with the issuance of a Joint Statement during Harper’s visit to China in December 2009. In 2012, Canada signed the Foreign Investment Promotion and Protection Agreement with China, and Toronto became the first RMB trading hub in North America in 2015. Despite increased economic cooperation as bilateral trade reached $85.9 billion in 2015, the Harper government sided with the USA and passed on the opportunity to become a founding member of the Asia Infrastructure Investment Bank.

After winning the federal election on October 20, 2015, Justin Trudeau’s government publicly stated its intention to strengthen its relationship with China—especially in the areas of trade and investment and collaboration on environmental issues—marking a new “golden decade” in China–Canada relations, as announced by Premier Li Keqiang during his trip to Canada in September 2016. In the meantime, growing isolationism in Washington further spurred Canada’s diversification strategy to reduce its dependence on the USA. As Trump slapped tariffs on Canadian steel and aluminum, pulled out of the Paris Agreement, and started renegotiating the North American Free Trade Agreement, the Trudeau government began exploratory talks for a Canada–China Free Trade Agreement (FTA), which would promise to increase Canadian exports to China by almost $7.7 billion and Canadian GDP by approximately $7.8 billion by 2030.

Against this backdrop, the first of the three surveys was conducted between August 30 and September 12, 2017, a few months before Trudeau’s December visit to Beijing. Many observers at the time believed that the trip would launch formal talks on the FTA, but these sky-high expectations fell short when Trudeau came back empty-handed, as Beijing refused to bundle the FTA with labor, gender, and environmental rights and protection. Things then took a turn for the worse in the next 12 months, which saw Ottawa being pulled into the vortex of a deepening geo-political and technological tug-of-war between the USA and China; this culminated in the arrest of Huawei CFO Meng Wanzhou in Vancouver in December 2018 at the request of the USA, and the subsequent arrest and detention of two Canadians in China.

The second survey was conducted between February 4 and 19, 2019, on the eve of the opening in a Vancouver courtroom of the judicial extradition hearing for Meng Wanzhou, when Canada–China relations had plunged to a historical low point since Tiananmen Square in June 1989. At the time, media commentary on China was overwhelmingly angry and critical in Canada. Developments in Xinjiang and Hong Kong, plus increased attention to Chinese influence and interference activities in Canada and elsewhere, further reinforced this negativity and led to calls for a more adversarial approach to China, parallel to Washington’s strategic competition with Beijing.

In the ensuing months, the diplomatic brawl remained at an impasse as the Meng case dragged on, while China launched a series of retaliatory measures, including an embargo on Canadian beef, pork, and canola oil worth billions of dollars, and rather spectacularly, for over 3 months, between June and September 2019, the ambassadorial positions in both countries were left unfilled. Conservative critics and China hawks in the government took this opportunity to openly criticize the Liberal government’s China policy, calling for new restrictions on trade, investment, and technological exchange. The last of the three surveys was implemented between September 26 and October 3, 2019, a few weeks before the federal election.

All three surveys were implemented via Qualtrics to a random sample of Canadian adults (age 18+). The sample sizes were 1519, 1161, and 1503, respectively. The respondents were sampled to match the latest census data on age, gender, and region. In the 2017 survey, for example, the average age in the sample is 46, compared to 41 for the national average. The gender ratio (number of males per 100 females) in the sample is 98, compared to 97 in the national statistics. The majority of the respondents are from Ontario (38%), followed by Québec (24%), British Columbia (18%), and Alberta (9%), similar to the population breakdown in the 2016 national census. Overall, the samples are representative of the entire adult population of Canada with respect to key demographic variables.

The surveys in September 2017 and October 2019 posed the same 60-plus substantive questions probing Canadian views on trade and investment issues, global leadership, Sino–USA comparisons, military and security matters, policy priorities, the state of human rights in China and how best to advance them, protecting Canadian values and institutions at home, information sources, and factors that shape views of China. The February 2019 survey asked a small subset of the questions from the full surveys. Many questions in these surveys replicate those posed in surveys conducted by other organizations, including Pew Research Center, the Lowy Institute, the Asia Pacific Foundation of Canada (APFC), and Angus Reid, to facilitate comparisons, but what sets these surveys apart from the others is their comprehensiveness. Furthermore, the timings of the surveys, at both high and low points in the bilateral relations, make it possible to evaluate whether and how far reactions to a current crisis shape longer-term views about China, the nature of the Canada–China relationship, and policy preferences.

## Public Perceptions of China in Canada Beyond the Feeling Thermometer

**Fig. 2:**
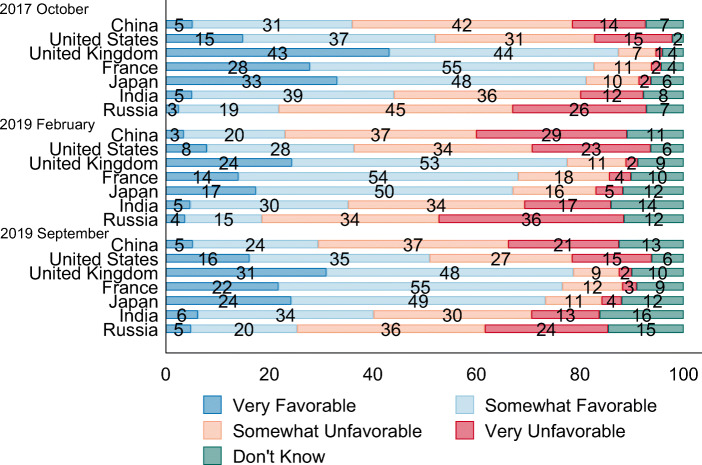
Canadian views on select countries. Source: Author’s survey

The analyses in this section focus on those questions that were asked in all three surveys. The first one is about favorability, which is measured on a four-point scale in response to the question “Please tell me if you have a very favorable, somewhat favorable, somewhat unfavorable or very unfavorable opinion of the following countries?” The countries used in all three surveys were China, the USA, the United Kingdom, France, Japan, India, and Russia. The results for all three surveys are plotted in Fig. 2. In October 2017, 36% of Canadians held favorable views on China and 56% held unfavorable views. These figures are better than for Russia and on par with India but lower than for the rest of the surveyed countries.

In February 2019, the favorability index for China experienced a substantial drop, with only 21% having a favorable opinion and 66% unfavorable. These numbers are almost identical to the Pew Global Attitudes Survey conducted around the same time (Silver et al. 2019). This cooling of opinions needs to be put into context, though, as most countries rated in the survey suffered a similar magnitude of reduction in favorability, with favorable views on the USA dropping even further, from 52 to 36%. In September 2019, public sentiments on China remained cool but had recovered slightly from 6 months earlier, with 29% of Canadians holding favorable views and 58% unfavorable.

These large shifts in public perceptions of China are consistent with the ups and downs in the bilateral relations at the time each survey was conducted. Even during the short-lived “golden era” of Canada–China relations, Canadians were not particularly fond of China, but the diplomatic crisis of the Meng Wanzhou case and its aftermath have soured their opinions of Beijing even more. Consistent with the pattern seen earlier in American opinions on China, this suggests that public perceptions of China abstracted to a single metric of favorable versus unfavorable can be highly sensitive to major shocks in bilateral relations.

Despite the overall unfavorable views, Canadians still considered China to be one of the most vital countries for Canada’s future. Table 1 displays the average ratings on an 11-point scale from least important (0) to most important (10) for the same seven countries across the three surveys in response to the question “In your view, how important are the following countries to Canada’s future?” Not surprisingly, the USA was perceived to be the most important country for Canada’s future, but China was not far behind, ranking second in 2017, ahead of the United Kingdom in third place. In the next two surveys, China’s overall standing fell to third, behind the USA and the UK, though it was virtually tied with the latter if we take into account the variability in the responses. As with the previous questions, however, the bigger context is that all countries experienced reductions in the absolute values of their importance in February 2019, then regained some of the losses in September 2019.

**Table 1:** Importance to Canada’s future

Countries	2017 October	2019 February	September 2019
USA	8.4	6.8	7.6
China	7.0	5.8	6.3
United Kingdom	6.6	6.0	6.4
Japan	5.9	5.5	5.9
France	5.4	5.0	5.7
India	5.3	4.6	5.0
Russia	4.7	4.1	4.6

Higher scores indicate more importance. Countries are ranked in descending order based on their importance in 2017. Source: Author’s survey

The next question asked respondents to compare China with the USA on a range of leadership questions, and the results are displayed in Table 2. Each cell represents the percentage of respondents that chose the respective country. In the 2017 survey, when favorability toward China was the highest, there was a visible lack of confidence about the role of the USA. While the USA was seen as the more responsible leader, more committed to freedom of speech, and respectful of people around the world for the next decade, three times as many respondents believed that China would become the biggest economic power in the world within the next decade. More respondents also perceived China as more likely to maintain peace, be more stable and predictable, and do more to address climate change and the environment.^1^

**Table 2:** Comparing China and the USA

	2017 October		2019 February		2019 September	
In the next decade, which country will be…	China	USA	China	USA	China	USA
the largest economic power?	64	21	52	26	47	33
the more responsible global leader?	28	36	22	36	21	43
doing more to maintain peace?	38	27	26	31	23	39
the more stable and predictable?	41	32	30	36	28	42
the more respectful of people around the world?	31	33	26	34	24	38
the more committed to freedom of speech?	8	69	10	61	9	65
doing more to address environmental issues?	36	30	26	31	22	41

Not shown are percentages of respondents who were “unsure.” Source: Author’s survey

In the February 2019 survey, China’s relative leadership potential fell in all categories except for the perception that China would be the biggest economic power in the world, though the latter was down to a two-to-one margin from just 18 months earlier. Most of China’s losses were accounted for by increases in the “do not know” responses rather than through a comparable rise in faith in the US leadership. These results largely mirrored those from the September 2019 survey for China, though confidence in the USA had increased across all seven categories.

The next set of questions probed public perceptions for more policy-relevant issues with respect to the Canadian government’s top priority on China. In the 2017 survey, as reported in Table 3, a third of the respondents (34%) believed that expanding trade and investment should be the federal government’s highest priority with China, followed by cooperating on global issues such as climate change, epidemics, and counter-terrorism (25%), protecting Canadian values and institutions at home from growing Chinese influence (15%), advancing human rights and democratic reforms in China (13%), and protecting cyber security (4%) and intellectual property rights (3%).

**Table 3:** Canada’s top priority on China

The government’s top priority on China	2017 October	2019 February	September 2019
Trade and investment	34	27	27
Cooperation on global issues	25	16	24
Protecting Canadian values	15	17	15
Human rights in China	13	14	11
Cyber security	4	9	8
Intellectual property rights	3	4	3
Do not know	5	13	12

Issues are listed in descending order based on their rankings in 2017. Cells may not add up to 100 due to rounding. Source: Author’s survey

In February 2019, public perceptions about the Canadian government’s top priority when engaging with China had remained largely consistent, with slight increases in promoting human rights and protecting Canadian values and institutions, and decreases in expanding trade and investment and furthering cooperation on global issues. The rankings were virtually unchanged, with the exception of those for cooperating on global issues and protecting Canadian values, which swapped positions for second and third places. The same can be said for results in the September 2019 survey, with the rankings reverting back to those from 2 years earlier.

One interesting finding is that advancing human rights and democratic reform in China is consistently perceived to be of much lower priority than trade and investment, among other things. This contrasts with the Angus Reid poll, which found that a majority of Canadians view human rights as more important than trade when given only these two options (Angus Reid Institute 2019). Again, this suggests that public preferences can be more diverse when people are not forced into making a choice between two diametrically different options.

Overall, these surveys reveal public attitudes to be more nuanced than a single measure of favorability can indicate. As the bilateral relationship between the two countries plunged to historic lows, public mood in Canada grew wary and cool, as expected. Moving from the general impression to specific policy areas, it becomes apparent that Canadians nonetheless wish to keep doors open to China, especially with respect to trade and investment. These seemingly conflictual preferences in public opinion persist when the policy areas are further narrowed down to specific policy issues, as will be seen in the case of Chinese FDI in the next section.

## Public Misperceptions About and Support for Chinese FDI in Canada

Often regarded as a vehicle for China’s global influence (Kastner and Pearson 2021), the recent influx of Chinese investments in every corner of the globe has raised concerns and even fear in the host countries. From the US regulators, scuttling deals made by Chinese investors in American tech start-ups to the UK’s review of a proposed Chinese-funded nuclear plant, politicians, business elites, and local communities are increasingly voicing their opposition to FDI from China, questioning motivations, business models, and socio-economic consequences for the host country.

The rise of Chinese investment has sparked similar debates in Canada, culminating in China National Offshore Oil Corporation’s bid to acquire Nexen, a Calgary-based Canadian oil and gas company, in 2012. Public discourse was divided over the proposed $15 billion mergers, with “private investor, financial sector, and media advocates of property rights on one side, and a broad cross-section of corporate executives and market-oriented economists on the other” (Hale 2014). Though the deal was approved after a lengthy review process, it led to the tightening of Canada’s FDI regulatory framework on future takeovers of Canadian firms by foreign state-owned enterprises.

Evidently, when Canadians said they welcome more economic exchange, they were not particularly enthusiastic about inviting more investments from China. According to a poll conducted by the APFC in 2015, Canadian views on Chinese FDI were more mixed than on FDI from other Asian countries and the USA, with “loss of control over resources,” “poor labor standards,” “environmental damage,” and “security risks” cited as major concerns (APFC 2015).

The 2017 survey confirmed these sentiments in a conjoint experiment, an increasingly popular tool in studies of politics and international political economy (see, e.g., Bechtel and Scheve 2013; Hainmueller and Hopkins 2014; Li and Zeng 2017).^2^ The conjoint experiment began with a short introductory text and instructions on how to complete the choice tasks. After this, respondents were asked to choose one of two hypothetical FDI proposals that would result in a minimum 10% change in shares of the ownership. Both proposals were associated with three attributes—country of origin, sector of investment, and ownership type of the foreign investor—which were presented side by side in a table.^3^ This binary comparison was repeated twice for every respondent, and each time, they were asked to pick the proposal they preferred.

**Fig. 3:**
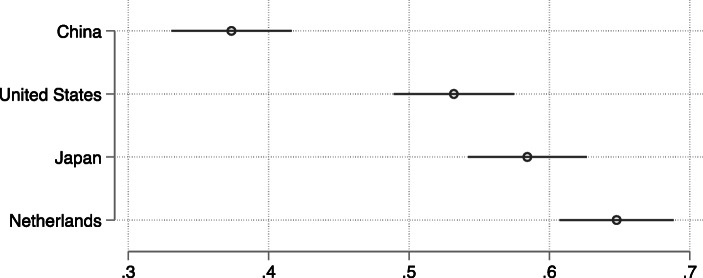
Preferences for FDI projects from different countries. The predicted probabilities are calculated from a linear probability model in Appendix B

Figure 3 plots the predicted probabilities that a respondent would pick an FDI project from each of the four countries, using estimates from a linear probability model that controls for other features of the FDI project and for individual-level characteristics of the respondents (see Appendix B for the full results). Projects from China were the least liked, with only 37% of the respondents favoring them over comparable ones from other countries, all of which received majority support.

Consistent with the findings from Kerry Ratigan (2021)’s study in Peru, features of the FDI project and demographic features such as age and party affiliation are significant predictors of the likelihood of disapproval for Chinese FDI,^4^ but as suggested by Haifeng Huang (2021) in this special issue, public misperceptions can be contributing factors as well. Scholars have documented prevalent misperceptions, blind spots, topical obsessions, and methodological missteps among China watchers in the USA (Li 2011; Johnston and Shen 2015). When it comes to investments from a new and unfamiliar investing country that traditionally has been the largest recipient of FDI, the tendency for misunderstanding can be substantial. Indeed, the APFC survey found that Canadians who significantly overestimated the extent of Chinese ownership in the Canadian economy were also more likely to say Canada has allowed “too much” investment from China to enter the country (APFC 2015).

The 2017 survey similarly documented widespread misperceptions regarding Chinese investments. Before the conjoint experiment discussed above, each respondent answered two questions regarding Chinese FDI in Canada that tapped into misperceptions arising from both innumeracy—i.e., the inability of ordinary citizens to process quantitative information (Peters 2006)—and misinformation, namely false and unsubstantiated beliefs about factual matters (Nyhan and Reifler 2010). The first question asked respondents to estimate the percentage of FDI stocks in Canada owned by Chinese firms. The second question asked respondents whether the following three statements regarding FDI regulations in Canada were true or false: (1) foreign companies that invest in Canada are not subject to Canadian laws and regulations, which may lead to lower labor or environmental standards; (2) large foreign investments must be approved by the federal government before going ahead; (3) investments made by foreign state-owned enterprises go through the same screening and approval procedure as those made by private companies.

**Fig. 4:**
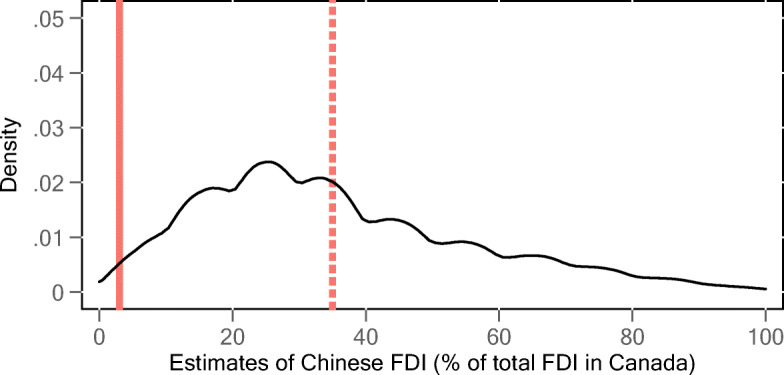
Estimates of Chinese FDI in Canada. The graph plots the distribution of respondents’ estimates about the percentage of FDI coming from China. The solid vertical line represents the official number from Statistics Canada, and the dashed vertical line is the average of the estimates from the respondents. Source: Author’s survey

Figure 4 plots the distribution of respondents’ estimates about the percentage of FDI coming from China. The solid line represents the official number from Statistics Canada, which is about 3%, and the dashed line is the average of the respondents’ estimates. These results confirm and measure public misperceptions about FDI. More specifically, respondents on average estimated that the percentage of FDI in Canada coming from China is 35%, which is similar to the estimates given by respondents in the APFC national poll and over ten times bigger than the Canadian official statistic of 3%. Only three out of the 1,519 respondents (3%) came up with the correct number.^5^

In terms of knowledge about the FDI regulatory framework in Canada, respondents did quite well with respect to the first two statements. On average, 70.3% and 80.6% of the respondents correctly identified that foreign companies must obey Canadian domestic laws and that large FDI projects need scrutiny and approval from the federal government before going ahead. These numbers are nearly identical to the numbers reported in the APFC survey. In contrast, Canadians scored much worse in the question regarding FDI from foreign SOEs: 19.3% gave the correct answer that these investments are subject to tighter rules and approval procedures set by the government. Altogether, only 163 out of the 1519 respondents answered all three questions correctly.

Does correcting misperceptions lead to changes in a respondent’s attitude toward FDI from China? Immediately after respondents estimated the proportion of Chinese FDI and answered the questions on FDI rules and regulations, half of them were randomly chosen to see the correct answers.^6^ This setup placed respondents into four groups. The first treatment group corrected the innumeracy of the respondents regarding the size of Chinese FDI in Canada relative to FDI from other major countries. The second treatment group corrected the misperception of the respondents regarding the rules and regulations governing FDI in Canada. Respondents in the third treatment group received both corrections, and the control group received neither.

**Fig. 5:**
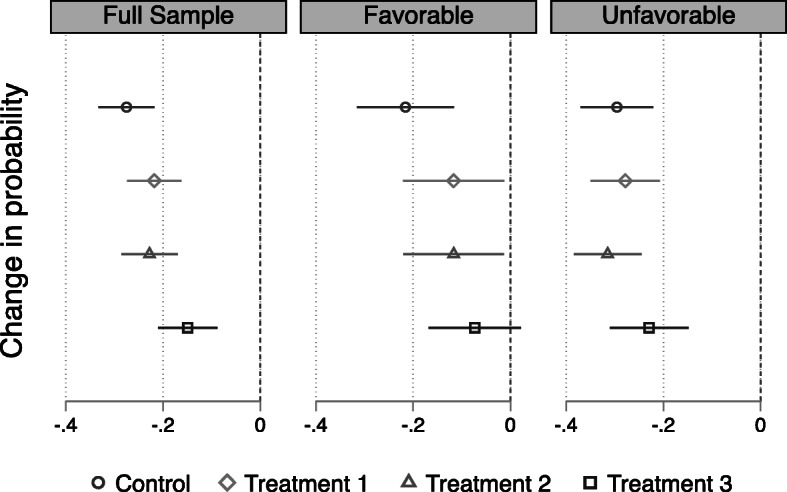
Effects of correcting Canadian misperceptions of Chinese FDI. This plot presents the change in the probability that an individual chooses an FDI project from China than one from the USA, Japan, or the Netherlands. All estimates in this figure are based on linear probability models, with whether or not a project is chosen as the dependent variable and FDI project features as independent variables. The bars denote 95% confidence intervals based on robust standard errors clustered by the respondent

The left panel of Fig. 5 illustrates the average treatment effects of correcting the misperceptions for the full sample. In the control group, where no correction was given, respondents were 27.5% less likely to favor FDI projects from China. Informing the respondents of either the correct amount of Chinese FDI in Canada (treatment 1) or Canada’s regulations governing foreign aid (treatment 2) narrowed the gap by about 5%, though the differences are not statistically significant. For respondents who received both corrections, however, the difference dropped further to 15%, which is equivalent to a statistically significant improvement in the odds ratio by 70%. Put differently, even though respondents still viewed Chinese FDI projects less favorably than comparable ones from Japan, the Netherlands, or the USA, receiving both corrections led to a sizable reduction in this “China penalty,” though not enough to eliminate it completely.

Further examination of the data shows that the effects of corrective information are conditional on respondents’ perception of China. As can be seen in the middle panel of Fig. 5, for those holding a favorable view of China, the pattern is similar to the one we see above in the full sample, but stronger. In fact, respondents in the treatment group who received both types of corrective information were no less likely to pick the FDI project from China over a comparable one from the USA, Japan, or the Netherlands—the 95% confidence interval of the estimated coefficient crosses zero. On the other hand, correcting misperceptions did not have any effect on respondents who held an unfavorable view of China—all four confidence intervals overlap in the right panel of Fig. 5. These heterogenous effects suggest that providing “inconvenient facts,” in Huang (2021)’s words, can help reduce misperceptions but may not be enough to shift the opinions of those holding strong negative views about the subject of those misperceptions.

## Conclusion

As the world continued grappling with the global COVID-19 pandemic, a new poll released in Canada on May 12, 2020, showed that perceptions of China had experienced another free fall, with only 14% of Canadians saying they had a positive opinion of China, half as many as six months earlier (Angus Reid Institute 2020). These eye-popping numbers propelled a new wave of media frenzy calling for Canada to “stand up to China and protect its own citizens from intimidation at home” (Bramham 2020). Policy makers seemed to quickly pick up the cue, as many believed that Trudeau’s sudden change of course the following day—raising questions, for the first time, about China’s role in the global pandemic—was a direct response to the people’s demand to “get tougher on China” (Fisher 2020).

What I have shown in this article, however, suggests that we should exercise caution before jumping to conclusions based on affective measures of favorability, due to the finicality and oversimplification of such measures. Going beyond the feeling thermometer, a deeper dive into public attitudes and preferences in Canada has revealed a significant foundation of public support for living and cooperating with China rather than decoupling from it, even during what many considered to be the lowest point in Canada–China relations since Tiananmen. These results echo two recent public opinion surveys on China in Australia (Kassam 2019) and the United Kingdom (Chow et al. 2019)—Australians and Britons also hold generally unfavorable views of Beijing but take a similarly pragmatic approach to bilateral relations with China.

For policy makers, the practical implications are to refrain from knee-jerk reactions to sudden shifts in public sentiments for quick political gains, and to better grasp the nuances and long-term trends in public opinion. In the context of Canada–China relations, these seemingly conflictual public opinions on various issues regarding China suggest that policy makers in Ottawa would be better served by steering away from outright befriending or disengaging with Beijing, and instead crafting a China policy that focuses on cooperating whenever possible, pushing back when necessary, and addressing growing public anxieties about China’s presence, influence, and interference in Canada’s domestic affairs.

## Supplementary Information

ESM 1 (DOCX 396 kb)

## Footnotes

1 Similar questions in the April 2017 Abacus poll found that 61% of Canadians felt China was doing more than the USA to try to maintain peace and avoid conflict, 57% that China was a better example of what world leadership looked like, and 54% that it was more stable and predictable than the USA (Anderson and Coletto 2017).

2 See Appendix A for more details as well as screenshots of the experimental design.

3 To minimize primacy and recency effects, the ordering and contents of the attributes are fully randomized.

4 Details can be found in Appendix C.

5 In the survey, respondents also estimated the percentages of FDIs in Canada from the USA, Japan, India, and the Netherlands. In all four cases, the estimates were much closer to the actual amounts calculated using official figures from Statistics Canada.

6 Details about the corrective messages can be found in Appendix A.
