# Supplementary material for: More than Meets the Eye: Understanding Perceptions of China Beyond the Favorable–Unfavorable Dichotomy
Source: Stud Comp Int Dev. 2021 Feb 24;56(1):68–86. doi: 10.1007/s12116-021-09320-1 (PMC7903215; doi:10.1007/s12116-021-09320-1)
Supplement: Supplementary file 1 — (DOCX 396 kb) [file 12116_2021_9320_MOESM1_ESM.docx]

**Appendix**

Appendix A: Survey Experiment Design

1. Every respondent read the following introduction at the beginning of the experiment.


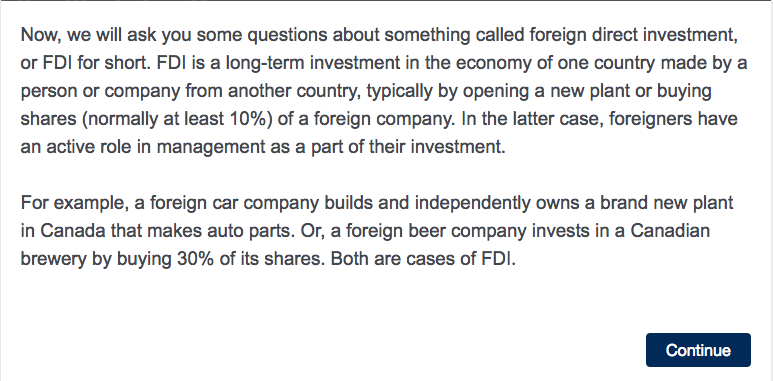


2. All respondents were asked to estimate the shares of FDI in Canada from five countries.


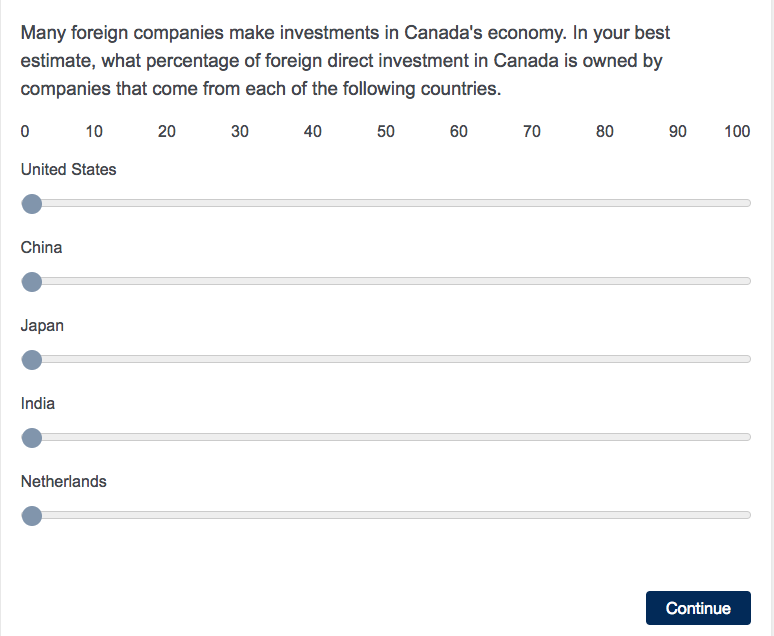


3. Respondents in the treatment group received the correct amounts. The column “your estimates” was filled with numbers from the previous question.


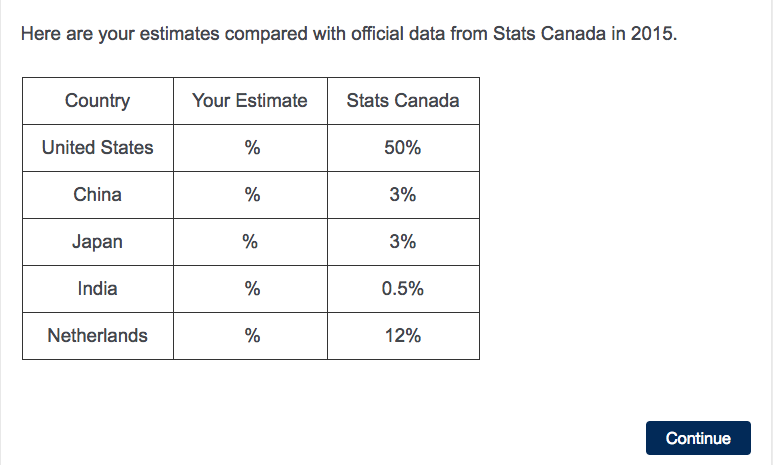


4. They also received the following question to ensure the correction worked.


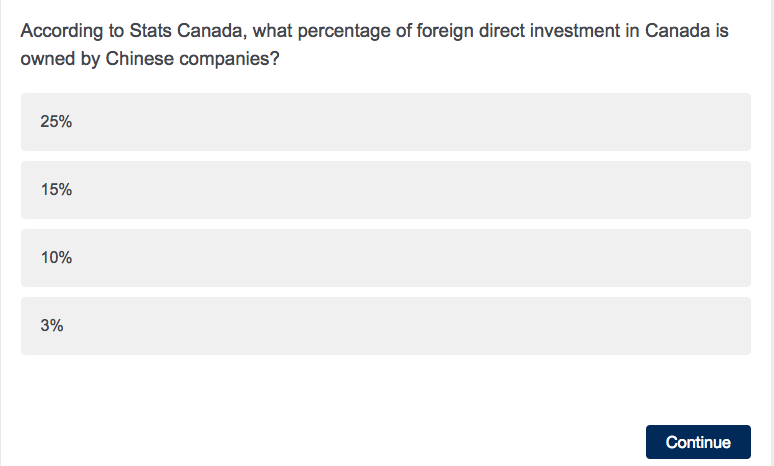


5. All respondents answered questions about the rules governing FDI. Respondents in the control group received all three statements on the same page.


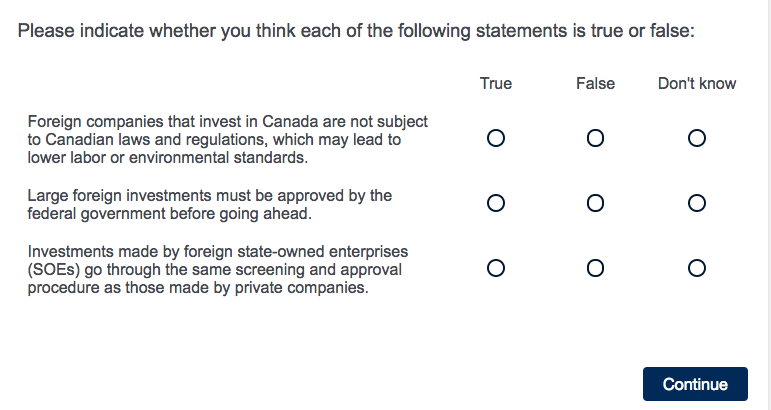


6. Respondents in the treatment groups saw the three statements one by one and were presented with the answer immediately after they answered each question. Depending on their choice, they saw one of the following:

If their answer was wrong: Oops, you got it **wrong**!

If their answer was right: Great, you got it **right**!

If their answer was “don’t know”: The correct answer is **false/true.**

They also were given a brief explanation:

According to**Global Affairs Canada** (formerly the Department of Foreign Affairs and International Trade), foreign investors in Canada **must abide by** Canadian domestic laws. In other words, foreign investors in Canada are not exempt from domestic competition laws or local regulations relating to **health, labor or the environment**.

According to the **Investment Canada Act (ICA)**, large foreign investments from private firms (over $600 million) and state-owned enterprises (over $379 million) **must be reviewed and approved** by the federal government before going ahead.

According to the **Investment Canada Act (ICA)**, in addition to the usual “net benefit” analysis, investments by state-owned enterprises, including sovereign wealth funds, are subject to **special guidelines** that involve broader and tighter checks on such criteria as the nature and extent of control by the foreign government, the SOE’s corporate governance, and its adherence to free-market principles.

7. All respondents answered the following question associated with the conjoint design. The table below is for illustration. Contents of the table (rows 2–4) were randomly chosen from a predetermined list of values.


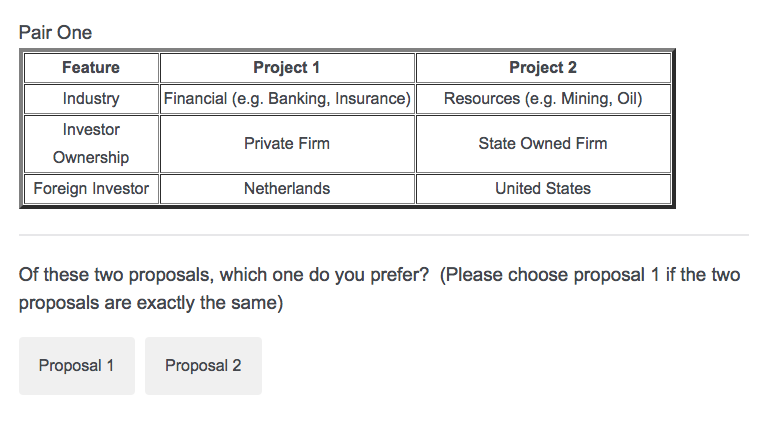


Appendix B: Linear Probability Model for Figure 3

|  |  |
| --- | --- |
| Dependent variable | Choosing the FDI project |
| *Features of the FDI project* |  |
| China | -0.274*** |
|  | (0.0188) |
| United States | -0.116*** |
|  | (0.0189) |
| Japan | -0.0637*** |
|  | (0.0182) |
| Natural resources | -0.104*** |
|  | (0.0202) |
| High-tech | 0.0794*** |
|  | (0.0193) |
| Financial | -0.0899*** |
|  | (0.0188) |
| State-owned firm | -0.0118 |
|  | (0.0135) |
| *Individual-level characteristics* |  |
| Male | -0.00185 |
|  | (0.00351) |
| Age | -7.04e-05 |
|  | (0.000121) |
| Education | -0.000477 |
|  | (0.00105) |
| Liberal Party | 0.00680* |
|  | (0.00397) |
| Conservative Party | 0.00298 |
|  | (0.00453) |
| Readership of news on China | 0.00326 |
|  | (0.00213) |
| Constant | 0.643*** |
|  | (0.0201) |
|  |  |
| Observations | 5,372 |
| R-squared | 0.061 |

Note: Robust standard errors clustered at individual respondents are in parentheses.

*** p<0.01, ** p<0.05, * p<0.1

Appendix C: Preference for Chinese FDI Projects as a Function of Project- and Individual-Level Features

|  |  |
| --- | --- |
| Dependent variable | Choosing the FDI project |
| *Features of the FDI project* |  |
| Natural resources | -0.635*** |
|  | (0.189) |
| High-tech | 0.309* |
|  | (0.170) |
| Financial | -0.454** |
|  | (0.180) |
| State-owned firm | -0.0206 |
|  | (0.126) |
| *Individual-level characteristics* |  |
| Male | 0.0918 |
|  | (0.119) |
| Age | -0.0170*** |
|  | (0.00445) |
| Education | 0.0126 |
|  | (0.0366) |
| Liberal Party | -0.277* |
|  | (0.142) |
| Conservative Party | -0.357** |
|  | (0.150) |
| Readership of news on China | -0.0802 |
|  | (0.0673) |
| Favorability toward China | 0.435*** |
|  | (0.0761) |
| Constant | 1.715*** |
|  | (0.428) |
|  |  |
| Observations | 1,196 |
| Log pseudo-likelihood | -723.71 |
| Pseudo R^2^ | 0.0530 |

Note: Robust standard errors clustered at individual respondents are in parentheses.

*** p<0.01, ** p<0.05, * p<0.1
